# Effect of Dysmetabolisms and Comorbidities on the Efficacy and Safety of Biological Therapy in Chronic Inflammatory Joint Diseases

**DOI:** 10.3390/jcm9051310

**Published:** 2020-05-02

**Authors:** Laura Cometi, Cosimo Bruni, Nicolò Chiti, Lorenzo Tofani, Francesca Nacci, Francesca Bartoli, Silvia Bellando-Randone, Daniela Melchiorre, Ginevra Fiori, Serena Guiducci, Marco Matucci-Cerinic

**Affiliations:** 1 Division of Rheumatology, Department of Experimental and Clinical Medicine, University of Florence, Via delle Oblate 4, 50141 Florence, Italy; cosimobruni85@gmail.com (C.B.); chiti.nico@gmail.com (N.C.); lorenzo120787@gmail.com (L.T.); clintrial@icloud.com (F.N.); francesca.bartoli19@gmail.com (F.B.); s.bellandorandone@gmail.com (S.B.-R.); daniela.melchiorre@unifi.it (D.M.); s.guiducci@hotmail.com (S.G.); marco.matuccicerinic@unifi.it (M.M.-C.); 2 Department of Geriatric Medicine, Division of Rheumatology Azienda Ospedaliera Universitaria Careggi, 50141 Florence, Italy; ginevrafiori@hotmail.com

**Keywords:** arterial hypertension, dyslipidemia, bDMARDs, rheumatoid arthritis, psoriatic arthritis, ankylosing spondylitis, adverse events, retention rate, angiotensin

## Abstract

In the present study we evaluated how systemic arterial hypertension (SAH), dyslipidemia and diabetes mellitus influence the efficacy, safety and retention rate of biological disease-modifying anti-rheumatic drug (bDMARD) treatment in rheumatic musculoskeletal disorders (RMDs). The charts of RMD patients treated with the first-line bDMARD were reviewed, collecting data on safety, efficacy and comorbidities at prescription (baseline, BL), after 6 months (6M) and at last observation on bDMARD (last observation time, LoT). In 383 RMD patients, a higher rate of adverse events at 6M (*p* = 0.0402) and at LoT (*p* = 0.0462) was present in dyslipidemic patients. Patients who developed dyslipidemia or SAH during bDMARD treatment had similar results (dyslipidemia *p* = 0.0007; SAH *p* = 0.0319) with a longer bDMARD retention as well (dyslipidemia *p* < 0.0001; SAH *p* < 0.0001). SAH patients on angiotensin converting enzyme inhibitors (ACEis) or angiotensin-II receptor blockers (ARBs) continued bDMARDs for longer than non-exposed patients (*p* = 0.001), with higher frequency of drug interruption for long-standing remission rather than inefficacy or adverse reactions (*p* = 0.0258). Similarly, dyslipidemic patients on statins had a better bDMARD retention than not-exposed patients (*p* = 0.0420). In conclusion, SAH and dyslipidemia may be associated with higher frequency of adverse events but a better drug retention of first-line bDMARD in RMDs, suggesting an additional effect of ACEis/ARBs or statins on the inflammatory process and supporting their use in RMD bDMARD patients with SAH/dyslipidemia.

## 1. Introduction

In patients with rheumatic musculoskeletal diseases (RMDs), biological disease-modifying anti-rheumatic drugs (bDMARDs) are currently an efficient therapeutic choice to achieve disease remission. It is well known that these drugs may interfere with glucose homeostasis, lipoprotein profile and systemic arterial blood pressure.

Data on systemic arterial hypertension (SAH) are controversial. A Japanese study demonstrated that infliximab reduced daytime blood pressure [1], while other data showed no effect of tumor necrosis factor inhibitor (TNFi) antibodies on blood pressure, neither systolic nor diastolic [2]. However, it was clear that TNFis increased levels of total cholesterol (TC), high-density lipoproteins (HDL), and, to a lesser extent, triglycerides (TG), without any effect on low-density lipoproteins (LDL) [3,4,5,6]. It was also shown that tocilizumab and tofacitinib increased TC and HDL levels [7]. Regarding glucose homeostasis, TNFi therapy reduced insulin levels and improved insulin resistance and insulin sensitivity [8].

There is currently no clear evidence on how alterations in glucose and lipid metabolism and SAH may affect the efficacy, safety and retention rate of bDMARDs. Conversely, data on obesity show that an elevated BMI has a negative influence on the response to TNFi therapy. In fact, obese patients achieve disease remission less frequently than non-obese patients [9,10].

The aim of this study was to evaluate the effect of SAH, dyslipidemia and diabetes on the efficacy, safety and retention rate of the first bDMARD in RMD, and to verify a possible effect of the concomitant medications used for the treatment of comorbidities.

## 2. Experimental Section

### 2.1. Materials and Methods

From January to August 2019, a retrospective review of the clinical charts of RMD patients followed in the Rheumatology Department (Azienda Ospedaliera Universitaria Careggi (AOUC)) from 2000 to 2018 was performed. Patients had a diagnosis of rheumatoid arthritis (RA), psoriatic arthritis (PsA) or ankylosing spondylitis (AS) (>18 years old) and were treated with the first on-label bDMARD for at least six months. Patients treated with bDMARDs for other off-label indications and patients whose medical records lacked data at least for the first six months of therapy were excluded. Data regarding bDMARDs lines from the second onwards were not evaluated in order to study a more homogeneous population and avoid possible confounding factors caused by the effects of previous bDMARDs. The study protocol and patients’ signed informed consent were approved by the local IRB (CAEVC protocol 15659).

Data were collected at baseline visit when bDMARD was prescribed (baseline, BL), at six months (6M) and at last visit (last observation time, LoT). The following data were collected:

- Anamnestic information: age, sex;
- Clinical data: age at diagnosis, diagnosis, inflammatory back pain, psoriasis, axial, entheseal or articular involvement, dactylitis, uveitis, inflammatory bowel diseases;
- Serological data: rheumatoid factor, ACPA, HLA-B27, CRP, ESR, TC, HDL, LDL, TG;
- Radiological features: edema or erosion on MRI;
- Previous and concomitant treatment: bDMARD, concomitant DMARDs, steroids, non-steroidal anti-inflammatory drugs (NSAIDs);
- Comorbidities and dysmetabolisms: dyslipidemia, SAH and diabetes and relative treatments;
- Efficacy data (assessment of the disease from the point of view of the patient and of the clinician, using a Likert scale on 3 points: 0 = worsening, 1 = stable, 2 = improvement of the disease compared to previous assessments).

At all visits, data about local and systemic adverse reactions (serious/not serious) were collected and divided into categories (infections, neoplasia, cardiovascular events, gastrointestinal events, bone marrow alterations, hepatic or renal function alteration, allergic events).

### 2.2. Statistical Analysis

Statistical analysis was performed with SAS 9.3 software (SAS Institute, Cary, NC, USA). The associations between categorical variables were tested by Chi-square test or by Fisher test, where appropriate; Spearman’s correlation test was used to investigate the correlations between non-parametric variables. The associations between continuous and categorical variables were evaluated by Student’s *t*-test or Mann–Whitney test, based on the distribution of the study variables. The continuous variables are presented as mean ± standard deviation, while categorical variables are presented as absolute frequency and percentage of each category. The duration of the bDMARD treatment was expressed by median and interquartile range. In all analyses, missing data were excluded. In all cases, the limit of statistical significance was set at *p* < 0.05.

## 3. Results

### 3.1. Study Population

Out of 1234 clinical charts, 77 (6.24%) were excluded due to missing data; 416 (33.71%) were treated with a bDMARD for other diseases other than RA, PsA or AS in off-label regimen; 31 (2.51%) patients lacked data for the first 6 months of therapy and 327 (26.50%) patients with RA, PsA or AS were not treated with a bDMARD. Therefore, 383/1234 (31.04%) patients were included in the analysis. The mean age was 51.67 ± 15.11 years, and the mean disease duration was 5.71 ± 7.02 years; 258 patients (67.36%) were females and 125 (32.64%) were males, with AS being the only RMD with male predominance (58.95%). In Table 1, the characteristics of the study population are reported and divided into the three RMDs.

Patients were stratified according to the bDMARD: 92% of patients received a TNFi as first bDMARD (33% etanercept, 32% adalimumab, 19% infliximab, 4% certolizumab, 4% golimumab) and the other 8% a non-TNFi bDMARD (3% tocilizumab, 2% abatacept, 2% rituximab, 1% ustekinumab). Table 2 reports RMD concomitant medications that patients received in association with bDMARDs at the BL visit.

Considering comorbidities, 99 patients (25.85%) suffered from SAH, 56 patients (19.38%) were affected by dyslipidemia and only 17 patients (4.54%) suffered from diabetes at the BL visit; these percentages were higher in the group of RA patients compared to the other diseases. The distribution of the comorbidities at baseline in the total cohort and stratified according to the disease are presented in Table 3, together with the new cases of each comorbidity registered at 6M and at LoT visit. 

### 3.2. Effect of Comorbidities on the Efficacy of First-Line Therapy with bDMARDs

At 6M and at LoT, we analyzed the effect of the comorbidities (SAH, dyslipidemia and diabetes) on the efficacy of bDMARDs. Patients affected by these comorbidities at baseline and those who developed them during therapy (before or after 6M visits but before LoT visits) were evaluated separately.

No significant influence of comorbidities on the efficacy of first bDMARD evaluated at the 6M visit was detected, both considering the patients who had these comorbidities at baseline and patients who developed these conditions over time before the 6M visit.

Regarding patients with new onset of the comorbidities during bDMARD therapy, the data show that SAH before the LoT visit was associated with higher rates of disease improvement when compared to non-hypertensive patients. This result was also confirmed considering the clinician evaluation (both 60.87% vs. 35.41%, *p* = 0.041).

### 3.3. Effect of Comorbidities on the Safety of First-Line Therapy with bDMARDs

Safety data analysis showed that patients who had dyslipidemia at the BL visit manifested a statistically significant higher rate of systemic adverse events both in the first six months of therapy (58.93% vs. 43.67%, *p* = 0.0402) and also later on, as reported at the LoT visit (80.36% vs. 66.67%, *p* = 0.0462). Analyzing the data of patients who developed these comorbidities during bDMARD treatment, a statistically significantly higher rate of systemic adverse events at the LoT visit was found for patients who developed dyslipidemia and SAH between the 6M and LoT visit when compared to patients who did not develop these conditions (dyslipidemia: 96.77% vs. 66.67%, *p* = 0.0007; SAH: 86.96% vs. 65.16%, *p* = 0.0319) (Table 4).

### 3.4. Effect of Comorbidities on the Retention Rate of First-Line Therapy with bDMARDs

The effect of dyslipidemia, SAH and diabetes on the retention rate of bDMARDs was analyzed and expressed in cumulative months of treatment, from BL to LoT visits.

From our data, patients who developed dyslipidemia or SAH during bDMARD treatment continued the drug for a longer period of time (dyslipidemia 95.48 months vs. 19.57 months, *p* < 0.0001; SAH 72.07 months vs. 23.40 months, *p* < 0.0001). No significant results were found for new-onset diabetes, although the number of cases was small (Table 5).

When repeating the analysis in the single disease groups, the same results were confirmed for RA and PsA (not for AS, probably due to the small number of patients in this group). In particular, for dyslipidemia in the RA group the retention was 100.63 months vs. 22.86 months (*p* = 0.0003) and in the PsA group it was 108.67 months vs. 19.64 months (*p* = 0.0016). Meanwhile, for SAH the retention was 61.74 months vs. 28.39 months in the RA group (*p* = 0.0188) and 80.49 months vs. 21.23 months in the PsA group (*p* = 0.0012) (Table 6).

### 3.5. Effects of Antihypertensive Therapy on Retention Rate of First-Line Therapy with bDMARDs

On the basis of the previously obtained results, we hypothesized that the effect of the comorbidities on the retention rate could be influenced by the treatments of these conditions. Therefore, we stratified patients based on antihypertensive treatment. Data showed that bDMARD-treated patients who concomitantly received therapy with ACE inhibitors and/or angiotensin II receptor blockers (ACEis/ARBs) continued the bDMARD for a longer period of time compared to nonACEi/ARB-exposed patients (40.53 months vs. 23.40 months, *p* = 0.001). Moreover, ACEi/ARB-treated patients more frequently maintained the therapy at LoT visit. In case of bDMARD interruption, this was due to well-being and disease remission rather than inefficacy or adverse reaction. Interruption due to inefficacy was more frequent in patients not treated with these drugs (*p* = 0.0258) (Table 7).

### 3.6. Effects of Statins on Retention Rate of First-Line Therapy with bDMARDs

Similar to ACEis/ARBs, data showed that patients concomitantly receiving statins for dyslipidemia continued the bDMARD for a longer period of time compared to patients who were not exposed to statins, including patients treated with other drugs and non-dyslipidemic patients (41.09 months vs. 26.50 months vs. 8.98 months, *p* = 0.0420). No statistically significant association between statin therapy and the reason of suspension at LoT visit was found.

## 4. Discussion

Our study showed that the presence of comorbidities such as SAH and dyslipidemia can influence the disease course in patients with inflammatory RMDs treated with bDMARDs. In particular, these comorbidities had an impact on the development of adverse reactions and the possibility of premature withdrawal. The data show that the presence of comorbidities had no significant influence on the efficacy of bDMARDs, from both patient and physician perspectives. Conversely, patients with dyslipidemia at BL or who developed dyslipidemia or SAH during bDMARD therapy developed much more systemic adverse events, mainly infectious complications. In addition, patients who developed SAH or dyslipidemia during bDMARD treatment continued biologic therapy for a longer period of time compared to the other patients. 

In particular, hypertensive patients who were concomitantly on ACEis/ARBs continued bDMARDs for a longer period of time than patients who were not on these drugs, and more frequently maintained the bDMARD at LoT visit. In the case of withdrawal in the ACEi/ARB-exposed cohort, this was due to well-being and disease remission rather than inefficacy or adverse reaction.

A possible explanation for these results relies on their pharmacological mechanisms of action. Both ACEis and ARBs interfere with the action of angiotensin II (AngII). It is well-known that AngII is also implicated in the inflammatory pathways, mediated by its binding to the AT1R (Angiotensin II Type 1 receptor). Therefore, the use of these anti-hypertensive drugs may help to improve inflammatory process control operated by the bDMARDs and the achievement of remission. In addition, by blocking the AT1R, ARBs make the circulating AngII stimulate the AT2R (Angiotensin II Type 2 receptor), which has anti-inflammatory action [11,12]. Moreover, we previously detected a marked expression of AT2R in fibroblast-like synoviocytes [13], and this might support the hypothesis of a direct effect of inflammation suppression at the articular level.

Previous attempts to treat RA with ACEis and ARBs have been reported. In RA, captopril alone improved symptoms and reduced acute-phase reactants in a slow but sustained way [14]. Additionally, valsartan reduced CRP and radical oxygen species (ROS) production in RA patients [15]. However, these studies were conducted on small cohorts of patients and without a direct comparison with traditional DMARDs. Therefore, even if the use of ACEis and/or ARBs cannot be supported as an RA treatment, they could represent the anti-hypertensive drug of choice in RA and, possibly, other inflammatory RMDs who present/develop SAH.

Similarly, our data showed that statins increased the retention of bDMARDs. This is in line with the anti-inflammatory properties of statins [16]. Moreover, the randomized-controlled TARA (Trial of Atorvastatin in Rheumatoid Arthritis) study demonstrated that the addition of atorvastatin to conventional treatment improved disease activity and reduced CRP levels as well as tender joint counts [17]. 

Our study has some strengths. This is the first study analyzing the impact of frequent comorbidities on bDMARDs’ efficacy, safety and retention rate in inflammatory RMDs. Moreover, our study population is widely representative of real-life cohorts, including different inflammatory RMDs and different bDMARDs’ mechanisms of action. Despite this, there are also some limitations; first of all, the observational design with a certain amount of missing data (mainly due to old clinical charts or to patients that had started the bDMARD in other rheumatologic centers and who unfortunately lacked data about the BL visit and the first months of treatment), which were handled with statistical adjustment. In particular, we had a great deal of missing information about dyslipidemia, as this condition was defined using laboratory parameters that were often lacking in the clinical charts. In addition, only a small number of patients had or developed diabetes mellitus, and therefore no conclusion could be drawn for this comorbidity. A possible explanation for the small incidence of diabetes in our cohort lies in the improvement of insulin sensitivity determined by TNFi. In fact, the study by Lillegraven et al. showed that exposure to TNFi was associated with a reduced incidence of diabetes with a HR of 0.35 (95% CI 0.13–0.91, *p* = 0.03) [18]. Finally, the observed population represents a sample of our bDMARD-treated RMD patients, with data collected and analyzed only for the first-line bDMARD therapy. This determined the inclusion of a great number of patients treated during the first decade of the century, when few bDMARD options were available (etanercept, adalimumab and infliximab). Future validation studies including the remaining bDMARD population in our center and investigating the effect of dysmetabolisms on second bDMARD lines onward are warranted.

## 5. Conclusions

In conclusion, our study showed that common comorbidities (i.e., SAH and dyslipidemia) have a significant impact on the safety and maintenance of bDMARDs for inflammatory RMDs. Moreover, results suggest a role of the renin–angiotensin–aldosterone axis in joint inflammation, as well as the possible use of ACEis and/or ARBs in association with traditional therapy to improve the control of disease activity. The use of statins in dyslipidemic patients may support the control of the inflammatory RMD activity. These data need to be confirmed in a larger cohort of patients to verify the real impact of extra-articular comorbidities and their specific therapies in patients with inflammatory RMD.

## Figures and Tables

**Table 1:** Characteristics of the study population.

	RA	PsA	AS	Missing Data
**Patients**	160 (41.78%)	128 (33.42%)	95 (24.80%)	-
**Age**	59.60 ± 13.58	49.10 ± 13.83	41.78 ± 11.97	0
**Disease duration**	6.96 ± 8.23	5.05 ± 5.74	4.57 ± 6.09	0
**Rheumatoid Factor positive**	93 (62.00%)	-	-	10
**Anti-CCP positive**	102 (68.46%)	-	-	11
**Psoriasis**	-	83 (65.87%)	13 (13.83%)	3
**Actual**	-	51 (61.44%)	7 (53.85%)	-
**Past**	-	14 (16.87%)	2 (15.38%)	-
**Familiar**	-	18 (21.69%)	4 (30.77%)	-
**Peripheral joint involvement**	-	112 (88.19%)	37 (38.95%)	1
**Entheseal involvement**	-	63 (49.61%)	43 (45.74%)	2
**Axial involvement**	-	46 (36.22%)	92 (96.84%)	1
**Dactylitis**	-	14 (10.93%)	6 (6.38%)	1
**Actual**	-	6 (42.86%)	4 (66.67%)	-
**Past**	-	8 (57.14%)	2 (33.33%)	-
**Nail psoriasis**	-	16 (12.50%)	1 (1.06%)	1
**Actual**	-	15 (93.75%)	1 (100.00%)	-
**Past**	-	1 (6.25%)	0 (0.00%)	-
**Uveitis**	-	2 (1.56%)	5 (5.26%)	0
**Actual**	-	0 (0.00%)	2 (40.00%)	-
**Past**	-	2 (100.00%)	3 (60.00%)	-
**Inflammatory back pain**	-	50 (39.37%)	92 (96.84%)	1
**Sacroiliac edema in MRI**	-	27 (60.00%)	44 (88.00%)	128
**Sacroiliac erosions in MRI**	-	2 (4.76%)	11 (23.40%)	134
**IBD**	-	-	22 (23.16%)	0
**HLA B27 positivity**	-	-	33 (40.24%)	13

RA: rheumatoid arthritis; PsA: psoriatic arthritis; AS: ankylosing spondylitis.

**Table 2:** Concomitant rheumatic musculoskeletal disease (RMD) medications in association with biological disease-modifying anti-rheumatic drugs (bDMARDs) at baseline (BL) visit.

	Total	RA	PsA	AS
**NSAIDs (n°, %)**	162 (42.29%)	46 (28.75%)	62 (48.43%)	54 (56.84%)
**DMARDs (n°, %)**	283 (73.89%)	144 (90.00%)	93 (72.65%)	46 (48.42%)
**methotrexate (n°, %)**	163 (42.56%)	96 (60.00%)	51 (40.16%)	16 (17.20%)
**leflunomide (n°, %)**	45 (11.75%)	26 (16.25%)	16 (12.60%)	3 (3.23%)
**sulphasalazine (n°, %)**	66 (17.23%)	7 (4.38%)	29 (22.83%)	30 (32.26%)
**hydroxychloroquine (n°, %)**	53 (13.84%)	47 (29.38%)	4 (3.15%)	2 (2.15%)
**steroids (predn. eq)**	3.23 ± 5.13	5.24 ± 5.61	2.30 ± 4.24	1.15 ± 4.15
**analgesics (n°, %)**	37 (9.66%)	13 (8.12%)	14 (10.94%)	11 (11.58%)

NSAIDs: non-steroidal anti-inflammatory drugs; RA: rheumatoid arthritis; PsA: psoriatic arthritis; AS: ankylosing spondylitis.

**Table 3:** Distribution of comorbidities in the study population at baseline (BL) and new cases of comorbidities at six months (6M) and last visit (LoT).

	Total (*n* = 383)	RA (*n* = 160)	PsA (*n* = 128)	AS (*n* = 95)	Patients with Missing Data (Total (RA; PsA; AS))
**SAH**	-	-	-	-	-
**BL**	99 (25.85%)	53 (33.13%)	31 (24.22%)	15 (15.79%)	0
**New 6M**	5 (1.32%)	4 (2.52%)	1 (0.79%)	0 (0.00%)	3 (1; 2; 0)
**New LoT**	23 (6.12%)	11 (7.01%)	10 (8.06%)	2 (2.11%)	7 (3; 4; 0)
**Dyslipidemia**	-	-	-	-	-
**BL**	56 (19.38%)	29 (22.66%)	22 (23.66%)	5 (7.35%)	94 (32; 35; 27)
**New 6M**	22 (10.73%)	12 (12.63%)	8 (11.43%)	2 (5.00%)	178 (65; 58; 55)
**New LoT**	31 (15.35%)	14 (15.05%)	6 (8.70%)	11 (27.50%)	181 (67; 59; 55)
**Diabetes**	-	-	-	-	-
**BL**	17 (4.45%)	14 (8.75%)	1(0.79%)	2 (2.11%)	1 (0; 1; 0)
**New 6M**	3 (0.80%)	1 (0.63%)	0 (0.00%)	2 (2.11%)	6 (2; 4; 0)
**New LoT**	1 (0.27%)	1 (0.64%)	0 (0.00%)	0 (0.00%)	9 (4; 4; 1)

New 6M: new cases of the comorbidity registered at the 6M visit; New LoT: new cases of the comorbidity at LoT visit. AS: ankylosing spondylitis; PsA: psoriatic arthritis; RA: rheumatoid arthritis.

**Table 4:** Effect of comorbidities (present at BL or developed during the treatment) on the development of adverse events (AEs) at 6M and LoT visits (the development of a comorbidity registered at the LoT visit was associated only with the development of AEs at the LoT visit, so the cells of the 6M visit are empty).

		Presence of AE at 6M (%)	*p*-Value	Presence of AE at LoT (%)	*p*-Value
**Comorbidity at BL**	-	-	-	-	-
**Dyslipidemia**	YES	58.93	**0.0402**	80.36	**0.0462**
	NO	43.67		66.67	
**SAH**	YES	41.24	0.3418	73.74	0.0942
	NO	46.81		64.54	
**Diabetes**	YES	35.29	0.3971	74.47	0.3883
	NO	45.98		66.39	
**New comorbidity at 6M**	-	-	-	-	-
**Dyslipidemia**	YES	31.82	0.0831	77.27	0.7424
	NO	51.37		74.03	
**SAH**	YES	40.00	0.8127	80.00	0.5243
	NO	45.31		66.49	
**Diabetes**	YES	33.33	0.6745	66.67	0.9922
	NO	45.45		66.40	
**New comorbidity at LoT**	-	-	-	-	-
**Dyslipidemia**	YES	-	-	96.77	**0.0007**
	NO	-		66.67	
**SAH**	YES	-	-	86.96	**0.0319**
	NO	-		65.16	
**Diabetes**	YES	-	-	100.00	0.4754
	NO	-		66.22	

**Table 5:** Effect of comorbidities (present at BL or developed during treatment) on the retention rate of the bDMARD treatment.

	Retention of bDMARD According to Comorbidity (Median (IR)), Months		*p*-Value
	Absence	Presence	-
**Comorbidity at BL**	**-**	**-**	**-**
**Dyslipidemia**	28.08 (11.91–72.07)	28.75 (15.61–69.61)	0.3566
**SAH**	27.60 (11.94–63.72)	24.88 (12.80–64.57)	0.9252
**Diabetes**	27.27 (12.01–64.18)	24.38 (13.62–52.07)	0.9252
**New comorbidity at 6M**	-	-	-
**Dyslipidemia**	29.49 (12.43–88.39)	23.85 (11.94–59.18)	0.6324
**SAH**	27.27 (12.04–63.68)	111.74 (20.23–118.13)	0.2341
**Diabetes**	26.94 (12.01–64.18)	44.24 (11.94–142.73)	0.4779
**New comorbidity at LoT**	-	-	-
**Dyslipidemia**	19.57 (9.97–56.45)	95.48 (60.13–134.87)	**<0.0001**
**SAH**	23.40 (11.94–60.49)	72.07 (32.60–117.93)	**<0.0001**
**Diabetes**	26.17 (11.99–64.14)	64.18 (64.18–64.18)	0.3909

Presence: presence of the comorbidity; Absence: absence of the comorbidity; IR: interquartile range.

**Table 6:** Effect of the development of dyslipidemia or systemic arterial hypertension (SAH) during the bDMARD treatment (registered at the LoT visit) on the retention rate of the bDMARD, stratified according to the disease.

	Retention of bDMARD According to Comorbidity (Median (IR)), Months		*p*-Value
	**Absence**	**Presence**	**-**
**Dyslipidemia at LoT**	**-**	**-**	**-**
**RA**	22.86 (9.51–59.18)	100.63 (60.13–130.86)	**0.0003**
**PsA**	19.64 (11.97–52.24)	108.67 (98.19–139.77)	**0.0016**
**AS**	14.34 (9.44–52.24)	66.32 (12.01–138.45)	0.0768
**SAH at LoT**	-	-	-
**RA**	28.39 (11.78–64.18)	61.74 (30.00–116.32)	**0.0188**
**PsA**	21.23 (11.97–49.24)	80.49 (40.53–118.98)	**0.0012**
**AS**	27.60 (11.94–64.57)	80.51 (22.57–138.45)	0.2906

Presence: presence of the comorbidity; Absence: absence of the comorbidity; IR: interquartile range; RA: rheumatoid arthritis; PsA: psoriatic arthritis; AS: ankylosing spondylitis.

**Table 7:** Association between outcome of biologic therapy at LoT visit and exposure to ACEis/ARBs (* Chi-Square test).

	ACE Inhibitors and/or AngII Receptor Blockers		
	NO	YES	*p*-Value
**Continuation of the drug**	125 (47.53%)	41 (59.42%)	0.0258 *
**Suspension due to wellness**	8 (3.04%)	5 (7.25%)	
**Suspension due to inefficacy**	86 (32.70%)	11 (15.94%)	
**Suspension due to adverse events**	44 (16.73%)	12 (17.39%)	

## Abbreviations

SAH	systemic arterial hypertension
bDMARD	biologic Disease Modifying Anti-Rheumatic Drugs
RMD	rheumatic musculo-skeletal disorders
BL	baseline visit
6M	6 month visit
LoT	last observation on bDMARD
ACEi	Angiotensin Converting Enzyme inhibitors
ARB	angiotensin II receptor blockers
TNFi	Tumor Necrosis Factor inhibitors
TC	total cholesterol
HDL	high-density lipoproteins
LDL	low-density lipoproteins
TG	triglicerides
BMI	body mass index
RA	rheumatoid arthritis
PsA	psoriatic arthritis
AS	ankylosing spondylitis
IRB	Institutional Review Board
ACPA	anti-citrullinated protein antibodies
HLA-B27	Human Leukocyte Antigen B27
CRP	C reactive protein
ESR	erythrocyte sedimentation rate
MRI	magnetic resonance imaging
NSAIDs	non-steroidal anti-inflammatory drugs
IBD	inflammatory bowel disease
AE	adverse event
IR	interquartile range
AT1R	angiotensinII receptor 1
AT2R	angiotensin II receptor 2
HR	hazard ratio
CI	confidence interval
